# Association of community engagement with vaccination confidence and uptake: A cross-sectional survey in Sierra Leone, 2019

**DOI:** 10.7189/jogh.12.04006

**Published:** 2022-02-26

**Authors:** Mohamed F Jalloh, Paul Sengeh, Ngobeh Ibrahim, Shibani Kulkarni, Tom Sesay, Victor Eboh, Mohammad B Jalloh, Samuel Abu Pratt, Nance Webber, Harold Thomas, Reinhard Kaiser, Tushar Singh, Dimitri Prybylski, Saad B Omer, Noel T Brewer, Aaron S Wallace

**Affiliations:** 1 Immunization Systems Branch, Global Immunization Division, U.S. Centers for Disease Control and Prevention, Atlanta, Georgia, USA; 2 FOCUS 1000, Freetown, Sierra Leone; 3 Expanded Program on Immunization, Ministry of Health and Sanitation, Freetown, Sierra Leone; 4 Epidemic Intelligence Service, U.S. Centers for Disease Control and Prevention, Atlanta, Georgia, USA; 5 Health Education Division, Ministry of Health and Sanitation, Freetown, Sierra Leone; 6 Sierra Leone Country Office of U.S. Centers for Disease Control and Prevention, Freetown, Sierra Leone; 7 Yale Institute of Global Health, Yale University, New Haven, Connecticut, USA; 8 Department of Health Behavior, Gillings School of Global Public Health, University of North Carolina, Chapel Hill, North Carolina, USA

## Abstract

**Background:**

The 2014-2016 Ebola epidemic disrupted childhood immunization in Sierra Leone, Liberia, and Guinea. After the epidemic, the Government of Sierra Leone prioritized community engagement to increase vaccination confidence and uptake. To support these efforts, we examined potential drivers of vaccination confidence and uptake in Sierra Leone.

**Methods:**

We conducted a population-based household survey with primary caregivers of children in a birth cohort of 12 to 23 months in four districts with low vaccination coverage in Sierra Leone in 2019. Modified Poisson regression modeling with robust variance estimation was used to examine if perceived community engagement in planning the immunization program in the community was associated with vaccination confidence and having a fully vaccinated child.

**Results:**

The sample comprised 621 age-eligible children and their caregivers (91% response rate). Half of the caregivers (52%) reported that it usually takes too long to get to the vaccination site, and 36% perceived that health workers expect money for vaccination services that are supposed to be given at no charge. When mothers were the decision-makers of the children’s vaccination, 80% of the children were fully vaccinated versus 69% when fathers were the decision-makers and 56% when other relatives were the decision-makers. Caregivers with high confidence in vaccination were more likely to have fully vaccinated children compared to caregivers with low confidence (78% versus 53%). For example, caregivers who thought vaccines are ‘very much’ safe were more likely to have fully vaccinated children than those who thought vaccines are ‘somewhat’ safe (76% versus 48%). Overall, 53% of caregivers perceived high level of community engagement, 41% perceived medium level of engagement, and 6% perceived low level of engagement. Perceiving high community engagement was associated with expressing high vaccination confidence (adjusted prevalence ratio (aPR) = 2.60; 95% confidence interval (CI) = 1.67-4.04) and having a fully vaccinated child (aPR = 1.67; 95% CI = 1.18-2.38).

**Conclusions:**

In these four low coverage districts in Sierra Leone, the perceived level of community engagement was strongly associated with vaccination confidence among caregivers and vaccination uptake among children. We have provided exploratory cross-sectional evidence to inform future longitudinal assessments to further investigate the potential causal effect of community engagement on vaccination confidence and uptake.

The 2014-2016 Ebola epidemic had a debilitating impact on health systems in Sierra Leone, Liberia, and Guinea [1,2]. High rates of Ebola infections among health workers [3] led to fear of contracting Ebola in health facilities and derailed confidence in health services, including immunization services [4]. In Sierra Leone, vaccination coverage of diphtheria-tetanus-pertussis vaccine (DTP-3) reached a high of 92% in 2013 before the Ebola epidemic but declined to 84% by the end of the health emergency in 2016 [5]. National vaccination coverage of the first dose of measles-containing vaccine (MCV-1) was estimated to be 81% based on survey data, but with substantial variation across districts [6]. Multiple outbreaks of measles occurred after the Ebola epidemic ended [7].

Beyond emergency contexts, caregivers may delay or refuse vaccines for their children due to complex individual, cultural, socioeconomic, and vaccination-related reasons [8-10]. Numerous community engagement efforts that involved working with affected communities to deeply understand issues and develop solutions were implemented in Sierra Leone during and after the Ebola epidemic to strengthen health systems and restore public confidence in health services, including childhood immunization [11-15]. Globally, major efforts are underway to strengthen community engagement to enhance confidence in childhood immunization [16], especially in low- and middle-income countries (LMICs) [17,18] and places recovering from disruptive health emergencies [19].

During and after major health emergencies, like the 2014-2016 Ebola epidemic and the current COVID-19 pandemic [20], childhood immunization services are often disrupted due to mitigation efforts such as restrictions in movement, fear of getting infected in clinics, and the shifting of limited resources to the health emergency [4]. In these emergency contexts, community engagement using dialogue, role modeling of promoted behaviors, and collective action planning is critical to safeguard confidence in immunization services, respond to misinformation about vaccines, and foster optimal vaccination uptake [21]. In Sierra Leone, we assessed potential drivers of vaccination uptake after the Ebola epidemic ended, including the potential role of perceived community engagement in planning the immunization program at the community level.

## METHODS AND MATERIALS

We conducted a population-based household survey with caregivers of children in a birth cohort of 12 to 23 months in four districts in Sierra Leone that was designed to allow the Sierra Leone Ministry of Health and Sanitation (MoHS) to understand drivers of vaccination and under-vaccination in low performing districts. These four districts had the lowest vaccination coverage of DTP-3 in their respective geographic regions (Kambia district in the Northern region, Kono district in the Eastern region, Moyamba district in the Southern region, and Western Rural in the Western region) [6]. We used multistage sampling to randomly select households, children, and caregivers. The sub-district sampling was done based on guidelines established by the World Health Organization [22]. The survey was conducted in February 2019.

### Setting

Sierra Leone has a civil society platform to strengthen community engagement in the planning and delivery of childhood immunization services [23]. The immunization civil society platform is an umbrella entity that comprises more than 100 organizations including community-based organizations, non-governmental organization (local and international), teachers associations, and local news media organizations. The Government of Sierra Leone included community engagement in the planning of health services as a core strategy in the post-Ebola recovery plan [24]. Priority activities included support for community-based networks to promote childhood immunization and to bring community stakeholders into the planning of immunization services [25], including through on-the-ground support from civil society. Moreover, community health workers (CHWs) have had a longstanding role in supporting childhood immunization in Sierra Leone, and were strongly leveraged in the post-Ebola recovery period [26].

### Measurement

The primary outcomes of interest were caregivers’ vaccination confidence and their children’s vaccination uptake. The primary exposure of interest was perceived community engagement in childhood immunization from the perspective of caregivers. To understand potential drivers of vaccination more comprehensively, we also assessed vaccination decision making, accessibility of childhood immunization services, preferred communication channels, and trusted communicators.

#### Perceived community engagement

We measured perceived level of community engagement by asking caregivers: “From your observation, how would you rate the level of community involvement and participation in the planning of vaccination programs in your community?” Response options were low, medium, or high.

#### Vaccination confidence

Items on vaccination confidence were informed by the Caregiver Vaccination Attitudes Scale that was validated in Ghana [27]. We used other theories to develop additional items on vaccination attitudes and experiences. For instance, perceived threat of vaccine-preventable disease and self-efficacy were informed by the Health Belief Model [28]. Additional items were developed to capture descriptive community norms around childhood vaccination and experiences during vaccination clinic visits. As part of a parallel urban needs assessment in Sierra Leone [29], we conducted cognitive testing of the confidence items via in-depth interviews with a convenience sample of five parents. Cognitive testing provided qualitative insights regarding how participants understood the questions and their interpretations of the response options. This process led to the finalization of 19 items on vaccination confidence that were included in the questionnaire.

#### Vaccination uptake

The questionnaire assessed vaccination uptake using the child’s home-based record (HBR) and through recall by the child’s primary caregiver when HBR was unavailable [22]. The child’s receipt of the following vaccines and related doses was assessed: Bacillus Calmette-Guerin (BCG) vaccine against tuberculosis; the first, second, and third dose of pentavalent vaccine (penta-1, penta-2, penta-3, respectively) against diphtheria, tetanus, pertussis, hepatitis B and *Haemophilus influenzae* type b; and MCV-1 against measles. Although the immunization schedule includes additional vaccines, the vaccine doses we assessed reflect the five scheduled immunization visits for infants in Sierra Leone – BCG at birth, penta-1 at 6 weeks, penta-2 at 10 weeks, penta-3 at 14 weeks, and MCV-1 at 9 months [26].

### Sampling

We randomly selected a total of 72 clusters (15 to 21 clusters per district) from a sampling frame of geographic enumeration areas based on the 2015 census [30]. Within each cluster, we randomly selected 10 eligible households and randomly selected one child from each household using simple random sampling via a mobile application. The corresponding primary caregiver was approached for their voluntary informed consent to an interview. We determined a target sample size of 720 caregiver-child pairs based on the following assumptions/parameters: 70% expected coverage of penta-3, desired precision of ±6.5%, α level of 0.05, and a design effect of 2.5.

### Training and data collection

We trained a local team that collected the data in local languages. The questionnaire was piloted in a conveniently selected community that was not part of the survey. Data collection took place in February 2019. A digital version of the questionnaire was programmed in Open Data Kit via KoboCollect version 1.2 (www.kobotoolbox.com) and installed on Android-based tablets.

### Statistical analysis

To reduce the 19-items on vaccination confidence into a conceptually meaningful scale, we conducted exploratory factor analysis (EFA) using a principal components extraction method. An oblique Promax rotation was applied to account for the correlation between factors [31]. We assessed the internal consistency reliability of the reduced items based on their combined Cronbach’s alpha coefficient. The mean score on the confidence scale was calculated and dichotomized; high confidence was defined as a score greater than the mean (coded 1) and low confidence was defined as having a score less than or equal to the mean (coded 0). For the vaccination uptake outcome, we created a composite binary variable wherein a ‘fully vaccinated’ child was defined as one who had received BCG, penta-1, penta-2, penta-3, and MCV-1; ‘under-vaccinated’ was defined as missing any of these vaccine doses.

We calculated proportions and 95% confidence intervals (CI) for point estimates in univariable and bivariable analyses, accounting for complex survey design. We examined the differences in point estimates between the caregivers of fully vaccinated and under-vaccinated children using a design-based *F* statistic that corrects the Pearson χ^2^ test for the complex survey design. Modified Poisson regression models with robust variance estimation [32,33] were fitted using generalized estimating equations with exchangeable correlation structure to account for the clustered data [34]. The use of modified Poisson regression modeling enabled us to directly calculate the adjusted prevalence ratios (aPR) [35].

In the first model, we examined the association between perceived community engagement (low, middle, or high) and expressing high vaccination confidence. In the second model, we examined the association between perceived community engagement and having a fully vaccinated child. We adjusted all models for the child’s birth order (coded 1 for the first child, 2 for the second child, 3 for at least the third child), where the child was delivered at birth (coded 1 for home-based delivery and 2 for delivery in a health facility), retention of the child’s vaccination card record (coded 0 for not retained and 1 for retained), parental education level (coded 0 if both parents had no education, 1 if one parent had some education, and 2 if both parents had some education), and parental religious affiliation (coded 1 if both parents were Christian, coded 2 if both parents were Muslim, coded 3 if parents had mixed-faith). These demographic covariates were included in the models because they have been shown to be consistently associated with vaccination uptake across diverse settings in West Africa and elsewhere [36,37]. All data were analyzed in Stata version 15 SE (StataCorp, College Station TX, USA). All statistical testing was two-sided and a *P*-value <0.05 was considered statistically significant.

### Ethical approval

The survey was approved by the Ethics and Scientific Review Committee in the Sierra Leone Ministry of Health and Sanitation. The Human Subject Office in the Center for Global Health at the U.S. Centers for Disease Control and Prevention approved the assessment as a non-research public health activity. All participants provided written or thumb-printed informed consent.

## RESULTS

**Figure 1:**
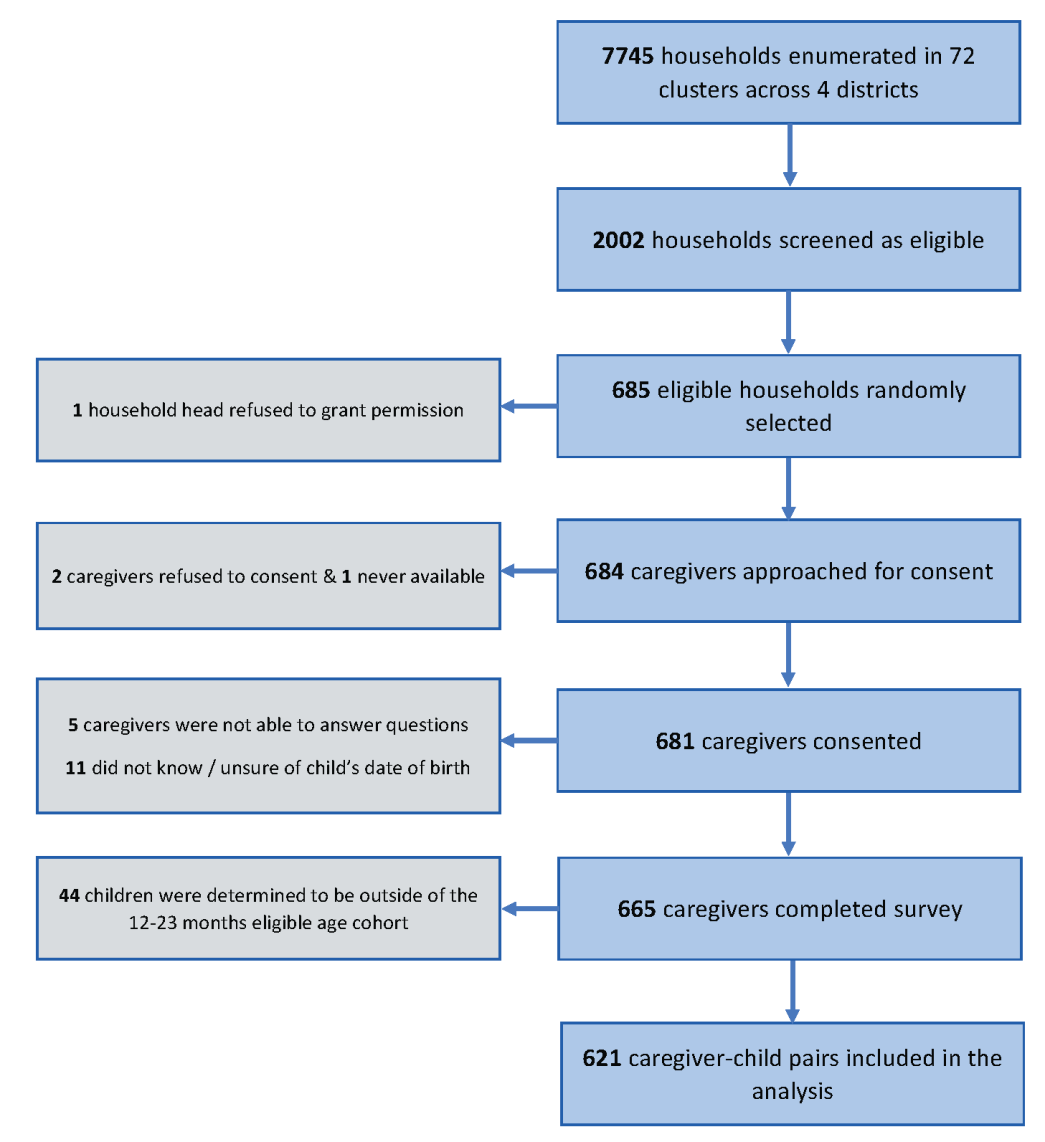
Flow diagram of the sampling of caregiver-child pairs included in the analysis–multistage cross-sectional cluster survey, Sierra Leone, 2019.

The final sample comprised of a birth cohort of 621 age-eligible children and their primary caregivers, which represented 91% of the 684 caregivers that were approached by the data collection teams (**Figure 1**). Nineteen percent of the children were delivered at home and 46% were at least the third child to the mother. Almost half of the mothers had some education (49%). Home-based vaccination card record was retained and available for 79% of the children (**Table 1**). Mothers were predominantly the primary caregivers of the children (90%), followed by siblings (5%), fathers (3%) and other relatives (2%) (data not shown in table).

**Table 1:** Sociodemographic characteristics of sampled children and their caregivers–multistage cross-sectional cluster survey, Sierra Leone, 2019

	All districts *N*=621, n (%)*	Kambia, *N*=140, n (%)*	Kono, *N*=161, n (%)*	Moyamba, *N*=156, n (%)*	Western Rural, *N*=164, n (%)*
**Child’s gender**					
Female	315 (51)	72 (51)	89 (55)	81 (52)	73 (45)
Male	306 (49)	68 (49)	72 (45)	75 (48)	91 (55)
**Child’s place of delivery**					
Home	120 (19)	27 (19)	12 (7)	44 (28)	37 (23)
Health facility	501 (81)	113 (81)	149 (93)	112 (72)	127 (77)
**Child’s birth order**					
First	211 (34)	37 (26)	37 (23)	71 (46)	66 (40)
Second	127 (20)	23 (16)	34 (21)	31 (20)	39 (24)
Third or greater	283 (46)	80 (57)	90 (56)	54 (35)	59 (36)
**Mother’s education**					
No education	314 (51)	88 (63)	84 (52)	88 (56)	54 (33)
Primary education	76 (12)	9 (6)	20 (12)	27 (17)	20 (12)
Secondary or higher	231 (37)	43 (31)	57 (35)	41 (26)	90 (55)
**Father’s education**					
No education	277 (46)	75 (55)	71 (46)	92 (61)	39 (25)
Primary education	31 (5)	2 (1)	18 (12)	8 (5)	3 (2)
Secondary or higher	292 (49)	59 (43)	64 (42)	52 (34)	117 (74)
**Mother’s occupation**					
Petty trader	250 (40)	49 (35)	68 (42)	23 (15)	110 (67)
Farmer	203 (33)	63 (45)	41 (26)	93 (60)	6 (4)
Private business	51 (8)	0 (0)	11 (7)	33 (21)	7 (4)
Student	45 (7)	16 (11)	10 (6)	5 (3)	14 (9)
Unemployed	44 (7)	10 (7)	15 (9)	2 (1)	17 (10)
Other	28 (5)	2 (1)	16 (10)	0 (0)	10 (6)
**Father’s occupation**					
Petty trader	66 (11)	12 (9)	6 (4)	3 (2)	45 (27)
Farmer	227 (37)	78 (56)	42 (26)	100 (64)	7 (4)
Private business	90 (15)	3 (2)	64 (40)	10 (6)	13 (8)
Student	30 (5)	14 (10)	4 (2)	5 (3)	7 (4)
Unemployed	19 (3)	2 (1)	6 (4)	1 (1)	10 (6)
Other	189 (30)	31 (22)	39 (24)	37 (24)	82 (50)
**Mother’s religion**					
Muslim	455 (73)	126 (90)	106 (66)	114 (73)	109 (66)
Christian	166 (27)	14 (10)	55 (34)	42 (27)	55 (34)
**Father’s religion**					
Muslim	470 (76)	129 (92)	113 (70)	115 (74)	113 (69)
Christian	151 (24)	11 (8)	48 (30)	41 (26)	51 (31)
**Retention of vaccination card**					
Not retained	131 (21)	0 (0)	32 (20)	50 (32)	49 (30)
Retained	488 (79)	140 (100)	128 (80)	106 (68)	114 (70)

*Unweighted frequencies and percent distributions of sample characteristics. Father’s education is missing 21 responses: 4 in Kambia, 8 in Kono, 4 in Moyamba, and 5 in Western Rural. Retention of vaccination card is missing 2 responses: 1 from Kono and 1 from Western Rural.

### Decision-making power related to vaccination

Most caregivers expressed that the child’s father held the final decision to vaccinate the child (56%), followed by the mother (37%) and other relatives (7%). In bivariable analysis, our findings suggest that the person who has the final decision-making power over the child’s vaccination was associated with vaccination uptake (*P*=0.04). For example, when mothers were the decision-makers, 80% (95% CI = 68%-89%) of the children were fully vaccinated versus 69% (95% CI = 60%-77%) when fathers were the decision-makers and 56% (95% CI = 38%-72%) when other relatives were (**Table 2**).

**Table 2:** Associations of decision making, accessibility, preferred information channels, and trusted communicators related to childhood immunization with having fully vaccinated children–multistage cross-sectional cluster survey, Sierra Leone, 2019

	Caregiver response		Child fully vaccinated†	
**N**	**% (95% CI)** *	**% (95% CI)** *	***P-*value** **‡**
**Person with final decision to vaccinate the child**				
Father	597	56 (49-65)	69 (60-77)	0.04
Mother		37 (30-46)	80 (68-89)	
Other relatives		7 (5-11)	56 (38-72)	
**Time usually taken to reach vaccination site**				
Too much time	620	52 (44-60)	73 (64-81)	0.80
About right		18 (11-28)	68 (44-85)	
Short time		30 (23-39)	70 (58-79)	
**Time usually spent waiting at vaccination site**				
Too much	620	59 (50-67)	73 (61-82)	0.67
About right		16 (12-22)	64 (47-78)	
Short time		25 (19-32)	73 (58-84)	
**Expected to give money to health workers**				
Some money	616	36 (27-47)	75 (61-85)	0.52
No money		64 (53-73)	69 (58-79)	
**Money expected by health workers is reasonable**				
Yes	193	62 (46-76)	78 (60-88)	0.56
No or unsure		38 (24-54)	71 (53-85)	
**Expected to give non-cash items to health workers**				
Yes	620	7 (4-11)	72 (59-82)	0.99
No or unsure		93 (89-96)	72 (64-79)	
**Non-cash items expected by health workers is reasonable**				
Yes	76	84 (66-94)	69 (55-81)	0.22
No or unsure		16 (7-34)	86 (56-97)	
**Most preferred communication channels**				
Health facilities	617	55 (43-67)	82 (74-88)	0.01
Community health worker household visits		25 (17-34)	62 (50-73)	
Radio programs		11 (7-17)	59 (48-70)	
Community-based events		7 (5-11)	48 (26-70)	
All other channels		2 (1-6)	36 (10-76)	
**Most trusted communicators**				
Health workers (eg, nurses)	617	67 (57-75)	76 (67-83)	0.15
Spouses		15 (11-21)	60 (48-71)	
Community health workers		8 (5-11)	62 (46-76)	
Child’s grandparents		3 (2-6)	56 (30-78)	
Others		7 (4-13)	71 (44-89)	

CI – confidence interval

*Proportions and 95% confidence intervals are weighted to account for the complex survey design.

†For analytical purposes, ‘fully vaccinated’ was defined as receiving BCG, penta-1, penta-2, penta-3, and MCV-1; under-vaccinated was defined as missing one or more of the aforementioned vaccine doses. Vaccination status was sourced from the home-based card record (HBR) for 488 children and from caregiver recall for 133 children whose HBRs were unavailable.

‡Due to the complex sampling design, to get a valid *P* value, the uncorrected *χ^2^* statistic was converted to an F statistic.

### Accessibility to vaccination services

Half of the caregivers (52%) reported that it took too much time to get to their usual vaccination site, and 59% expressed that too much time is spent waiting for the child to be vaccinated at the health facility. Caregivers perceived that health workers expected some money (36%) or non-cash items (7%) in return for vaccination. Among the sub-sample of caregivers who perceived some expectation of monetary or non-cash items (*n*=193), most of them expressed that they found the amount of money or non-cash items to be reasonable (62% and 84%, respectively). These aspects of immunization service accessibility were not found to be associated with vaccination uptake in bivariable analysis (**Table 2**).

### Preferred communication channels

The most preferred communication channels were through health facilities (55%) and household visits by CHWs (25%). In bivariable analysis, communication channel preference was associated with vaccination uptake (*P*=0.01). For example, caregivers who preferred getting immunization information from health facilities were more likely to have fully vaccinated children compared to caregivers who preferred radio programs (82%, 95% CI = 74%-88%, versus 59%, 95% CI = 48%-70%). 

### Trusted communicators

Health workers were largely viewed as the most trusted communicators of immunization information (67%), followed by spouses (15%), and CHWs (8%). Preference for trusted communicators was not associated with vaccination uptake (*P*=0.15) (**Table 2**).

### Perceived community engagement

Overall, 6% of respondents perceived low level of community engagement, 41% perceived a medium level of engagement and 53% perceived a high level of engagement. In bivariable analysis, perceived community engagement was associated with vaccination uptake (*P*<0.01). For example, children whose caregivers perceived high level of engagement were 82% (95% CI = 72%-88%) fully vaccinated compared to 63% (95% CI = 53%-71%) of children whose caregivers perceived medium level of engagement and 36% (95% CI = 22%-53%) of children whose caregivers perceived low level of engagement (**Table 3**).

**Table 3:** Associations of perceived community engagement, perceived areas of improvement, and measures of vaccination confidence with having fully vaccinated children – multistage cross-sectional cluster survey, Sierra Leone, 2019

	Caregiver response		Child fully vaccinated†	
**N**	**% (95% CI)** *	**% (95% CI)** *	***P-*value** **‡**
**Perceived level of community engagement**§				
High	602	53 (43-63)	82 (72-88)	<0.01
Medium		41 (32-50)	63 (53-71)	
Low		6 (4-10)	36 (22-53)	
**Perceived priority areas of improvement**				
More vaccination sites closer to the community	589	35 (24-47)	68 (54-79)	0.29
Enhance community engagement and education		32 (23-43)	68 (57-73)	
Improve vaccinator interactions with caregivers		11 (6-22)	77 (55-90)	
Reduce waiting time at vaccination sites		9 (6-13)	78 (64-88)	
Make vaccines safer		3 (1-7)	59 (40-75)	
Offer vaccination services on weekends		2 (1-4)	91 (67-98)	
Other improvements not listed above		6 (2-17)	91 (63-98)	
No improvements needed		2 (1-4)	72 (38-91)	
**Vaccines are good for the child**				
Very much	620	85 (78-91)	74 (67-81)	0.01
Somewhat		14 (8-25)	51 (34-69)	
Very little or not at all		1 (0-2)	47 (16-80)	
**Vaccines are safe for the child**				
Very much	619	83 (75-89)	76 (68-82)	<0.01
Somewhat		15 (10-23)	48 (37-59)	
Very little or not at all		2 (1-3)	44 (16-77)	
**Vaccines protect child against diseases**				
Very much	619	82 (73-89)	76 (68-83)	<0.01
Somewhat		16 (10-25)	49 (39-59)	
Very little or not at all		2 (1-4)	39 (18-65)	
**Confident in ability to take the child for vaccination**				
Very much	619	84 (75-90)	76 (68-83)	<0.01
Somewhat		14 (8-22)	49 (38-60)	
Very little or not at all		2 (1-5)	25 (10-49)	
**Encourage others to get their children vaccinated**				
Very much	619	80 (70-87)	77 (69-83)	<0.01
Somewhat		8 (4-13)	55 (36-72)	
Very little or not at all		12 (7-20)	45 (37-54)	
**People in the community value vaccination**				
Very much	618	78 (69-85)	77 (69-84)	<0.01
Somewhat		13 (9-18)	55 (43-67)	
Very little or not at all		9 (5-19)	44 (32-58)	
**Spouse or partner approves of vaccination**				
Very much	614	84 (75-90)	77 (69-83)	<0.01
Somewhat		10 (6-17)	49 (36-62)	
Very little or not all		6 (4-10)	40 (25-57)	
**Other parents in the community approve of vaccination**				
Very much	615	80 (71-87)	77 (69-83)	<0.01
Somewhat		14 (9-21)	56 (44-68)	
Very little or not at all		6 (3-9)	35 (21-53)	
**Trusted leaders in the community approve of vaccination**				
Very much	619	75 (65-83)	77 (68-84)	<0.01
Somewhat		18 (12-25)	55 (45-64)	
Very little or not at all		7 (4-11)	56 (40-71)	
**Religion influences vaccination decision for the child**				
Very much	618	73 (62-81)	77 (69-84)	<0.01
Somewhat		14 (10-21)	65 (52-76)	
Very little or not at all		13 (8-20)	47 (37-58)	
**Vaccination is compatible with religious beliefs**				
Very much	613	75 (66-82)	79 (71-85)	<0.01
Somewhat		13 (9-19)	51 (40-63)	
Very little or not at all		12 (8-19)	50 (38-62)	
**Measles is a health threat for unvaccinated children**				
Very much	619	85 (77-91)	76 (68-82)	<0.01
Somewhat		13 (8-20)	47 (36-59)	
Very little or not at all		2 (1-4)	3 (15-54)	
**Illnesses that vaccines prevent are severe**				
Very much	618	83 (74-89)	75 (67-83)	<0.01
Somewhat		14. (9-22)	49 (37-61)	
Very little or not at all		3 (2-5)	30 (13-55)	
**How people usually talk about vaccination in community**				
Mostly positive	569	77 (68-84)	79 (70-85)	<0.01
Mixed		22 (16-30)	57 (49-64)	
Mostly negative		1 (0-3)	24 (6-59)	
**Vaccination confidence scale** **‖**				
High confidence	546	78 (69-85)	78 (70-85)	<0.01
Low confidence		22 (15-31)	53 (41-64)	

CI – confidence interval

*Measured with a 3-point Likert item asking participants: “From your observation, how would you rate the level of community involvement and participation in the planning of vaccination programs in your community?” Response options were low, medium, or high.

†Proportions and 95% confidence intervals are weighted to account for the complex survey design.

**‡**For analytical purposes, ‘fully vaccinated’ was defined as receiving BCG, penta-1, penta-2, penta-3, and MCV-1; under-vaccinated was defined as missing one or more of the aforementioned vaccine doses. Vaccination status was sourced from the home-based card record (HBR) for 488 children and from caregiver recall for 133 children whose HBRs were unavailable.

§Due to the complex sampling design, to get a valid *P* value, the uncorrected χ2 statistic was converted to an F statistic.

‖The vaccination confidence scale score was dichotomized into a binary variable to indicate low vaccination confidence (≤ mean score) versus high vaccination confidence (> mean score).

### Perceived priority areas of improvement

About one third of respondents wanted more vaccination sites closer to the community (35%) and enhanced community engagement and education (32%). In addition, improving interactions between vaccinators and caregivers was cited by 11% of respondents. In bivariable analysis, perception of the priority areas of improvement was not associated with vaccination uptake (*P* = 0.29) (**Table 3**).

### Vaccination confidence

Psychometric properties of the Childhood Vaccination Confidence Scale used in the assessment are provided in Table S1, Table S2, and Table S3 in the **Online Supplementary Document**. In summary, the 19 items on vaccination confidence were reduced to a 12-item scale based on results from the exploratory factory analysis. Two factors were retained in the final scale, namely ‘confidence’ (10 items total) and ‘congruence with faith’ (2 items total) (Table S1 in the **Online Supplementary Document**). The 12-item scale demonstrated very good internal consistency (Cronbach’s α = 0.94). The scale score was positively associated with having a fully vaccinated child after adjusting for demographic covariates such that every 1 unit increase in the scale score was associated with a 26% increase in the likelihood of the child being fully vaccinated (Table S3 in the **Online Supplementary Document**). 

When the scale score was dichotomized, 78% of caregivers scored above the mean and were considered as having high confidence in vaccination. In bivariable analysis, among caregivers with high confidence in vaccination, 78% (95% CI = 70%-85%) of their children were fully vaccinated compared to 53% (95% CI = 41%-64%) among caregivers with low confidence (Table 3). For example, children whose caregivers thought vaccines are ‘very much’ safe were 76% (95% CI = 68%-82%) fully vaccinated compared to 48% (95% CI = 37%-59%) of children whose caregivers thought vaccines are ‘somewhat’ safe. Children whose caregivers thought vaccination is ‘very much’ compatible with their religious beliefs were 79% (95% CI = 71%-85%) fully vaccinated compared to 51% (95% CI = 40%-63%) of children whose caregivers thought vaccination is ‘somewhat’ compatible with their religious beliefs. Children whose caregivers felt that community discourse about vaccination is ‘mostly positive’ were 79% (95% CI = 70%-85%) fully vaccinated compared to 24% (95% CI = 6%-59%) of children whose caregivers felt it is ‘mostly negative’ and 57% (95% CI = 49%-64%) of children whose caregivers felt it is ‘mixed’ (**Table 3**).

### Vaccination uptake

About 98% of children in our sample received BCG, 97% received penta-1, 94% received penta-2, 81% received penta-3, and 84% received MCV-1. In total, 71% of the children in our sample were considered fully vaccinated and 29% were under-vaccinated. Self-reported vaccination refusal was low (3%) but self-reported vaccination delay was more frequently reported by caregivers (19%). Nearly all children received their last vaccine at a health facility (99%) compared to via community outreach (1%) (**Table 4**).

**Table 4:** Childhood vaccination uptake, self-reported refusal, and self-reported delay– multistage cross-sectional cluster survey, Sierra Leone, 2019

	Total sample		
	***N***	%*	95% CI*
**Vaccination uptake (based on home-based record only)†**			
Bacillus Calmette-Guerin vaccine	488	98	95.6-99.4
1^st^ dose of pentavalent vaccine	488	98	96.4-99.1
2^nd^ dose of pentavalent vaccine	488	95	91.5-97.2
3^rd^ dose of pentavalent vaccine	488	89	83.4-92.4
1^st^ dose of measles-containing vaccine	488	82	76.0-87.2
Received all vaccine doses (fully-vaccinated)	488	79	70.8-84.7
Missed one or more vaccine doses (under-vaccinated)	488	21	15.3-29.2
**Vaccination uptake (based on home-based record and caregiver recall)†**			
Bacillus Calmette-Guerin vaccine	618	98	96.0-99.1
1^st^ dose of pentavalent vaccine	618	97	94.7-98.6
2^nd^ dose of pentavalent vaccine	609	94	90.8-96.2
3^rd^ dose of pentavalent vaccine	609	81	75.2-85.5
1^st^ dose of measles-containing vaccine	619	84	79.4-88.1
Received all vaccine doses (fully-vaccinated)	619	71	63.5-77.9
Missed one or more vaccine doses (under-vaccinated)	619	29	22.1-36.5
**Self-report of any past refusal or delay of vaccination**			
Refused vaccination	620	3	1.8-5.3
Delayed vaccination	616	19	13.5-26.6
**Vaccination strategy used for most recent vaccine received**			
Static / fixed post (caregiver took child to a health facility)	620	99	98.8-99.9
Community outreach (vaccinator visited community/household)		1	0.1-1.2

CI – confidence interval

*Proportions and 95% confidence intervals are weighted to account for the complex survey design.

†Vaccination status was sourced from the home-based card record (HBR) for 488 children and from caregiver recall for 133 children whose HBRs were unavailable.

### Association between perceived community engagement and vaccination confidence

In multivariable analysis, caregivers who perceived high community engagement were nearly three times more likely to express high vaccination confidence (adjusted prevalence ratio (aPR) = 2.60; 95% CI = 1.67-4.04) as compared to caregivers who perceived low community engagement. A caregiver was more likely to express high vaccination confidence when both parents have some education versus no education (aPR = 1.19; 95% CI = 1.02-1.40). Other demographic covariates were not associated with vaccination confidence (**Table 5**).

**Table 5:** Multivariable correlates of expressing high vaccination confidence and having a fully vaccinated child – multistage cross-sectional cluster survey, Sierra Leone, 2019

	High vaccination confidence†		Fully vaccinated child‡	
**Adjusted PR§ (95% CI)**	***P*-value**	**Adjusted PR§ (95% CI)**	***P value‖***
Perceived community engagement*				
Low	Reference		Reference	
Medium	2.42 (1.59-3.71)	<0.01	1.38 (0.98-1.94)	0.06
High	2.60 (1.67-4.04)	<0.01	1.67 (1.18-2.38)	<0.01
**Child’s place of delivery**				
Home	Reference		Reference	
Health facility	1.12 (0.98-1.26)	0.09	1.28 (1.06-1.54)	0.01
**Child’s maternal birth order**				
First	Reference		Reference	
Second	0.96 (0.85-1.09)	0.57	1.08 (0.93-1.25)	0.34
Third or greater	0.97 (0.85-1.09)	0.58	1.13 (0.98-1.31)	0.10
**Parents’ education**				
No parent has any education	Reference		Reference	
One parent has some education	1.08 (0.95-1.24)	0.25	1.15 (0.97-1.35)	0.10
Both parents have some education	1.19 (1.02-1.40)	0.03	1.33 (1.16-1.53)	<0.01
**Parents’ religion**				
Both Christian	Reference		Reference	
Both Muslim	1.03 (0.97-1.16)	0.59	0.96 (0.82-1.11)	0.57
Mixed faith	0.98 (0.85-1.12)	0.77	1.17 (0.99-1.38)	0.07
**Retention of child’s vaccination card**				
Not retained	Reference		Reference	
Retained	0.98 (0.88-1.11)	0.79	1.82 (1.48-2.23)	<0.01

PR – prevalence ratio; CI – confidence interval

*Measured by asking participants: “From your observation, how would you rate the level of community involvement and participation in the planning of vaccination programs in your community?” Response options were low, medium, or high.

^†^The vaccination confidence scale score was dichotomized into a binary variable to indicate low vaccination confidence (≤ mean score) versus high vaccination confidence (> mean score).

^‡^ Defined as a child who received Bacillus Calmette-Guerin vaccine, three doses of pentavalent vaccine, and first dose of measles vaccine based on the home-based card record (HBR) for 488 children and from caregiver recall for 133 children whose HBRs were unavailable.

^§^PR, prevalence ratio from modified Poisson regression model with robust variance estimation by using generalized estimating equation; models adjusted for child’s place of delivery, child’s birth order, parents’ education, parents’ religion, retention of vaccination card.

^‖^Due to the complex sampling design, to get a valid *P* value, the uncorrected χ^2^ statistic was converted to an F statistic.

### Association between perceived community engagement and vaccination uptake

In multivariable analysis, caregivers who perceived high community engagement were nearly twice as likely to have children who were fully vaccinated compared to those who perceived low engagement (aPR = 1.67, 95% CI = 1.18-2.38). Demographically, a child was more likely to be fully vaccinated when the caregiver retained the child’s vaccination card versus not retaining the card (aPR = 1.82; 95% CI = 1.48-2.23), the child was delivered in a health facility versus at home (aPR = 1.28; 95% CI = 1.06-1.54), and when both parents have some education versus no education (aPR = 1.33; 95% CI = 1.16-1.53) (**Table 5**).

## DISCUSSION

Three years after major disruptions in the childhood immunization program that were precipitated by the 2014-2016 Ebola epidemic in Sierra Leone, nearly all caregivers in our assessment expressed that vaccines are good, safe, and beneficial in preventing diseases in children. In districts with low vaccination coverage, compared to caregivers of under-vaccinated children, those with fully vaccinated children were more likely to encourage vaccination, perceive religious compatibility with vaccination, feel respected by the vaccination staff, and prefer to receive immunization information from health facilities. In multivariable analysis, caregivers who perceived a high level of community engagement were nearly three times more likely to express high vaccination confidence and nearly twice as likely to have children that were fully vaccinated compared to caregivers who perceived low level of engagement. In addition, we validated the use of a vaccination confidence scale for the first time in Sierra Leone, which to our knowledge is also the first validated vaccination confidence scale in a low-income country [38].

Dropout between vaccine doses is a major contributor of under-vaccination in LMICs [39] that propagates health inequities [40]. In these settings, community engagement efforts that make caregivers feel that their communities are *listened to* in the planning of the childhood immunization program may boost confidence and motivate completion of the infant immunization schedule. Examples of community engagement activities in the Sierra Leonean context includes use of civil society organizations to engage in dialogue with community leaders to get them to serve as advocates for immunization in their communities; leveraging of religious leaders to promote the benefits of immunization through sermons; organizing community outreach activities jointly with CHWs and immunization staff to identify and vaccinate children who have missed scheduled vaccine doses, especially in geographically hard-to-reach areas [23].

Community engagement is increasingly acknowledged as a critical component to build vaccination confidence and achieve optimal population-level vaccination uptake, including for vaccines outside of the routine childhood immunization program such as COVID-19 vaccination. However, quantifiable evidence of the causal effects of community engagement to promote immunization remains limited globally [41-46]. A randomized control trial in India has been registered to examine the impact of community engagement on vaccination uptake (43), but results are not yet available. In addition, there is a published protocol for a systematic review on the use of community engagement to increase vaccination uptake in LMICs (42), but without any published results as of date. In the interim, our present results provide cross-sectional evidence showing that community engagement is strongly associated with vaccination confidence and vaccination uptake in Sierra Leone. Our findings are consistent with a descriptive observational study that showed increases in vaccination uptake following enhanced community engagement in Ethiopia (41). Although there is no one-size-fits-all approach to community engagement, the lack of standardized principles and approaches has made it challenging to rigorously evaluate and compare the effects of community engagement within and across LMICs. In 2020, UNICEF developed a set of minimum quality standards for community engagement that can be used to guide the development and evaluation of community engagement interventions [47]. Future assessments of community engagement in immunization programs would benefit from adapting these standard indicators to enable cross-country comparisons of the effects of specific attributes of community engagement on immunization outcomes.

Constraints in accessing immunization services have been documented in urban areas in Sierra Leone [29] and other settings in sub-Saharan Africa [48]. In our assessment, bringing additional vaccination sites closer to the community was recommended as the most important intervention area to improve childhood immunization in the community. One way to operationalize this recommendation is by conducting more frequent community outreach sessions to reach under-vaccinated children. A separate assessment in Sierra Leone uncovered that community outreach sessions were not conducted four times monthly as recommended by WHO for the African region, and that CHWs were underutilized in defaulter-tracking efforts [49]. Enhancing the frequency and quality of the outreach visits and using community engagement principles may help improve vaccination confidence and uptake.

Despite governmental efforts to remove cost barriers for all caregivers through the Free Healthcare Initiative in Sierra Leone, our assessment showed that payments to health workers during vaccination visits is a common expectation among caregivers. While this practice may discourage caregivers who are unable to pay and compromise the trust in health workers [50,51], in our sample, we did not find any differences in vaccination uptake between caregivers who expected to provide informal payments versus those who did not expect to do so. A prior assessment in Sierra Leone found that informal payments may be precipitated by under-staffed health facilities needing to pay ‘volunteer’ health workers who are not formally on the government’s payroll [52]. Involving communities in the planning of the immunization program and instituting mechanisms to sustainably get community feedback may contribute to ensuring accountability in service delivery, and therefore boost trust in and ownership of the services [51].

### Limitations

Our study has a few limitations. First, all measures were based on self-report. Implementation of various health promotion activities in these districts may have prompted socially desirable responding. Related, we do not have verifiable data on actual implementation of community engagement activities promoting childhood immunization in these districts. Finally, we cannot infer a causal relationship between the perceived level of community engagement and vaccination confidence and uptake in our cross-sectional study.

## CONCLUSIONS

Our assessment identified a range of potential drivers of childhood immunization after a complex health emergency in four districts in Sierra Leone. We found that caregivers who perceived high level of community engagement were nearly three times more likely to express high vaccination confidence and their children were nearly twice as likely to be fully vaccinated. The exploratory cross-sectional evidence we have provided may inform future longitudinal assessments to investigate the potential causal effect of community engagement on vaccination confidence and uptake.

## Additional material


Online Supplementary Document

## References

[1] Elston JW Cartwright C Ndumbi P Wright J The health impact of the 2014-15 Ebola outbreak. Public Health. 2017;143:60-70. 10.1016/j.puhe.2016.10.020 28159028

[2] Sun X Samba TT Yao J Yin W Xiao L Liu F Impact of the Ebola outbreak on routine immunization in western area, Sierra Leone - a field survey from an Ebola epidemic area. BMC Public Health. 2017;17:363. 10.1186/s12889-017-4242-7 28446173 PMC5406892

[3] Kilmarx PH Clarke KR Dietz PM Hamel MJ Husain F McFadden JD Ebola virus disease in health care workers–Sierra Leone, 2014. MMWR Morb Mortal Wkly Rep. 2014;63:1168-71. 25503921 PMC4584541

[4] Yerger P Jalloh M Coltart CEM King C Barriers to maternal health services during the Ebola outbreak in three West African countries: a literature review. BMJ Glob Health. 2020;5:e002974. 10.1136/bmjgh-2020-002974 32895217 PMC7476472

[5] World Health Organization. WHO-UNICEF estimates of DTP3 coverage. 2019. Available: https://apps.who.int/immunization_monitoring/globalsummary/timeseries/tswucoveragedtp3.html. Accessed: 21 June 2019.

[6] UNICEF. Sierra Leone Multiple Indicators Cluster Survey. 2017. Available: https://mics.unicef.org/news_entries/106/SIERRA-LEONE-2017-MICS-RELEASED. Accessed: 21 January 2021.

[7] Masresha BG Luce R Jr Weldegebriel G Katsande R Gasasira A Mihigo R The impact of a prolonged ebola outbreak on measles elimination activities in Guinea, Liberia and Sierra Leone, 2014-2015. Pan Afr Med J. 2020;35:8. 10.11604/pamj.supp.2020.35.1.19059 32373259 PMC7196330

[8] Dubé E Gagnon D MacDonald NE Strategies intended to address vaccine hesitancy: Review of published reviews. Vaccine. 2015;33:4191-203. 10.1016/j.vaccine.2015.04.041 25896385

[9] Dubé E Laberge C Guay M Bramadat P Roy R Bettinger J Vaccine hesitancy: an overview. Hum Vaccin Immunother. 2013;9:1763-73. 10.4161/hv.24657 23584253 PMC3906279

[10] Brewer NT Chapman GB Rothman AJ Leask J Kempe A Increasing Vaccination: Putting Psychological Science Into Action. Psychological science in the public interest. Psychol Sci Public Interest. 2017;18:149-207. 10.1177/1529100618760521 29611455

[11] United Nations Development Programme. Recovering from the Ebola Crisis - Full Report. 2017. Available: https://www.undp.org/content/undp/en/home/librarypage/crisis-prevention-and-recovery/recovering-from-the-ebola-crisis—full-report.html. Accessed: 21 January 2021.

[12] Marston BJ Dokubo EK van Steelandt A Martel L Williams D Hersey S Ebola Response Impact on Public Health Programs, West Africa, 2014-2017. Emerg Infect Dis. 2017;23:S25. 10.3201/eid2313.170727 29155674 PMC5711323

[13] Alpren C Jalloh MF Kaiser R Diop M Kargbo S Castle E The 117 call alert system in Sierra Leone: from rapid Ebola notification to routine death reporting. BMJ Glob Health. 2017;2:e000392. 10.1136/bmjgh-2017-000392 28948044 PMC5595198

[14] Blevins JB Jalloh MF Robinson DA Faith and Global Health Practice in Ebola and HIV Emergencies. Am J Public Health. 2019;109:379-84. 10.2105/AJPH.2018.304870 30676797 PMC6366492

[15] Jalloh MF Wilhelm E Abad N Prybylski D Mobilize to vaccinate: lessons learned from social mobilization for immunization in low and middle-income countries. Hum Vaccin Immunother. 2020;16:1208-14. 10.1080/21645515.2019.1661206 31464551 PMC7227704

[16] World Health Organization. Improving vaccination demand and addressing hesitancy. 2020. Available: http://awareness.who.int/immunization/programmes_systems/vaccine_hesitancy/en/. Accessed: 18 January 2021.

[17] Hub VD. Behaviorally Informed Interventions. 2021. Available: https://www.demandhub.org/behaviorally-informed-interventions/. Accessed: 21 January 2021.

[18] Shea B Andersson N Henry D Increasing the demand for childhood vaccination in developing countries: a systematic review. BMC Int Health Hum Rights. 2009;9 Suppl 1:S5. 10.1186/1472-698X-9-S1-S5 19828063 PMC3226237

[19] UNICEF. UNICEF Immunization Roadmap 2018-2030. 2018. Available: https://www.unicef.org/sites/default/files/2019-01/UNICEF_Immunization_Roadmap_2018.pdf. Accessed: 21 January 2021.

[20] Outbreak Observatory. The impact of COVID-19 on routine immunization. 2020. Available: https://www.outbreakobservatory.org/outbreakthursday-1/12/10/2020/w9hy6szcowmz14zc8npyml92x9k8mb. Accessed: 7 January 2021.

[21] Jalloh MF Nur AA Nur SA Winters M Bedson J Pedi D Behaviour adoption approaches during public health emergencies: implications for the COVID-19 pandemic and beyond. BMJ Glob Health. 2021;6:e004450. 10.1136/bmjgh-2020-004450 33514594 PMC7849902

[22] World Health Organization. Vaccination Coverage Cluster Surveys. 2015. Available: https://www.who.int/immunization/monitoring_surveillance/Vaccination_coverage_cluster_survey_with_annexes.pdf. Accessed: 5 June 2018.

[23] Catholic Relief Services. Civil Society Organization Platforms Contribute to National Immunization Programs. 2019. Available: https://www.crs.org/sites/default/files/tools-research/promising_practices_a4_final_rev071119_online.pdf. Accessed: 4 April 2021.

[24] Government of Sierra Leone. National Ebola Recovery Strategy for Sierra Leone. 2015. Available: https://ebolaresponse.un.org/sites/default/files/sierra_leone_-_national_recovery_strategy_2015-2017.pdf. Accessed: 5 December 2020.

[25] Gavi. Proposal for HSS support (2017-2021): Sierra Leone. 2017. Available: https://www.gavi.org/sites/default/files/document/2021/Proposal%20for%20HSS%20support%282017-2021%29-Sierra%20Leone.pdf. Accessed: 4 April 2021.

[26] Government of Sierra Leone. Comprehensive EPI Multi-Year Plan 2017-2021. 2016. Available: https://www.gavi.org/sites/default/files/document/2021/cMYP%20Sierra%20Leone%202017-2021.pdf. Accessed: 5 December 2020.

[27] Wallace AS Wannemuehler K Bonsu G Wardle M Nyaku M Amponsah-Achiano K Development of a valid and reliable scale to assess parents’ beliefs and attitudes about childhood vaccines and their association with vaccination uptake and delay in Ghana. Vaccine. 2019;37:848-56. 10.1016/j.vaccine.2018.12.055 30642731 PMC6534746

[28] Glanz KR, B.K.; Viswanath, K. Health beahvior: Theory, research, and practice. Fifth edition ed. San Francisco, California: Jossey-Bass; 2015 July 2015.

[29] Feldstein LR Sutton R Jalloh MF Parmley L Lahuerta M Akinjeji A Access, demand, and utilization of childhood immunization services: A cross-sectional household survey in Western Area Urban district, Sierra Leone, 2019. J Glob Health. 2020;10:010420. 10.7189/jogh.10.010420 32509292 PMC7243070

[30] Statistics SierraLeone. 2015 Sierra Leone Population and Housing Census. 2016. Available: https://www.statistics.sl/index.php/census/census-2015.html. Accessed: 12 November 2020.

[31] Thompson B. Exploratory and confirmatory factor analysis: Understanding concepts and applications. Washington DC: American Psychological Association; 2004.

[32] Zou G A modified poisson regression approach to prospective studies with binary data. Am J Epidemiol. 2004;159:702-6. 10.1093/aje/kwh090 15033648

[33] Zou GY Donner A Extension of the modified Poisson regression model to prospective studies with correlated binary data. Stat Methods Med Res. 2013;22:661-70. 10.1177/0962280211427759 22072596

[34] Yelland LN Salter AB Ryan P Performance of the modified Poisson regression approach for estimating relative risks from clustered prospective data. Am J Epidemiol. 2011;174:984-92. 10.1093/aje/kwr183 21841157

[35] Barros AJ Hirakata VN Alternatives for logistic regression in cross-sectional studies: an empirical comparison of models that directly estimate the prevalence ratio. BMC Med Res Methodol. 2003;3:21. 10.1186/1471-2288-3-21 14567763 PMC521200

[36] Kazungu JS Adetifa IMO Crude childhood vaccination coverage in West Africa: Trends and predictors of completeness. Wellcome Open Res. 2017;2:12. 10.12688/wellcomeopenres.10690.1 28459105 PMC5407439

[37] Tauil MC Sato AP Waldman EA Factors associated with incomplete or delayed vaccination across countries: A systematic review. Vaccine. 2016;34:2635-43. 10.1016/j.vaccine.2016.04.016 27109562

[38] Shapiro GK Kaufman J Brewer NT Wiley K Menning L Leask J A critical review of measures of childhood vaccine confidence. Curr Opin Immunol. 2021;71:34-45. 10.1016/j.coi.2021.04.002 34000455 PMC10932019

[39] World Health Organization. 20 million children miss out on lifesaving measles, diphtheria and tetanus vaccines in 2018. 2019. Available: https://www.who.int/news/item/15-07-2019-20-million-children-miss-out-on-lifesaving-measles-diphtheria-and-tetanus-vaccines-in-2018. Accessed: 1 January 2021.

[40] World Health Organization. Immunization coverage. 2020. Available: https://www.who.int/news-room/fact-sheets/detail/immunization-coverage. Accessed: 15 January 2021.

[41] Demissie SD Kozuki N Olorunsaiye CZ Gebrekirstos P Mohammed S Kiapi L Community engagement strategy for increased uptake of routine immunization and select perinatal services in north-west Ethiopia: A descriptive analysis. PLoS One. 2020;15:e0237319. 10.1371/journal.pone.0237319 33119604 PMC7595373

[42] Jain ME. M.; Gaarder, M.; Bagai, A.; Eyers, J. PROTOCOL: Use of community participation interventions to improve child immunisation in low- and middle-income countries: A systematic review and meta-analysis. 2020. Available: https://onlinelibrary.wiley.com/doi/full/10.1002/cl2.1119. Accessed: 29 January 2021. 10.1002/cl2.1119 PMC8356293 37016605

[43] Lassi ZS Das JK Salam RA Bhutta ZA Evidence from community level inputs to improve quality of care for maternal and newborn health: interventions and findings. Reprod Health. 2014;11 Suppl 2:S2. 10.1186/1742-4755-11-S2-S2 25209692 PMC4160921

[44] Lukusa LA Mbeye NN Adeniyi FB Wiysonge CS Protocol for a systematic review of the effects of interventions to inform or educate caregivers about childhood vaccination in low and middle-income countries. BMJ Open. 2015;5:e008113. 10.1136/bmjopen-2015-008113 26169807 PMC4513514

[45] Pramanik S Ghosh A Nanda RB de Rouw M Forth P Albert S Impact evaluation of a community engagement intervention in improving childhood immunization coverage: a cluster randomized controlled trial in Assam, India. BMC Public Health. 2018;18:534. 10.1186/s12889-018-5458-x 29688845 PMC5913885

[46] Saeterdal I Lewin S Austvoll-Dahlgren A Glenton C Munabi-Babigumira S Interventions aimed at communities to inform and/or educate about early childhood vaccination. Cochrane Database Syst Rev. 2014;11:CD010232. 10.1002/14651858.CD010232.pub2 25408540 PMC10880811

[47] UNICEF. Minimum quality standards and indicators in community engagement. 2020. Available: https://www.unicef.org/mena/reports/community-engagement-standards. Accessed: 1 August 2020.

[48] Vasudevan L Baumgartner JN Moses S Ngadaya E Mfinanga SG Ostermann J Parental concerns and uptake of childhood vaccines in rural Tanzania - a mixed methods study. BMC Public Health. 2020;20:1573. 10.1186/s12889-020-09598-1 33081744 PMC7573867

[49] Jalloh MF Namageyo-Funa A Gleason B Wallace AS Friedman M Sesay T Assessment of VaxTrac electronic immunization registry in an urban district in Sierra Leone: Implications for data quality, defaulter tracking, and policy. Vaccine. 2020;38:6103-11. 10.1016/j.vaccine.2020.07.031 32753291 PMC10869104

[50] Hsiao A Vogt V Quentin W Effect of corruption on perceived difficulties in healthcare access in sub-Saharan Africa. PLoS One. 2019;14:e0220583. 10.1371/journal.pone.0220583 31433821 PMC6703670

[51] Lewis M Informal payments and the financing of health care in developing and transition countries. Health Aff (Millwood). 2007;26:984-97. 10.1377/hlthaff.26.4.984 17630441

[52] Witter S Wurie H Bertone MP The free health care initiative: how has it affected health workers in Sierra Leone? Health Policy Plan. 2016;31:1-9. 10.1093/heapol/czv006 25797469 PMC4724164

