# Supplementary material for: Association of community engagement with vaccination confidence and uptake: A cross-sectional survey in Sierra Leone, 2019
Source: J Glob Health. 2022 Feb 26;12:04006. doi: 10.7189/jogh.12.04006 (PMC8876869; doi:10.7189/jogh.12.04006)
Supplement: Online Supplementary Document [file jogh-12-04006-s001.pdf]

Association of community engagement with vaccination confidence and uptake: A cross-sectional survey in Sierra Leone, 2019

SUPPLEMENTARY MATERIAL

Table S1. Item means and factor structure of the 12-item Childhood Vaccination Acceptance and Confidence Scale–Sierra Leone, 2019

	N*	Mean* (SD)	Factor loading†	
			Confidence	Congruence with faith
Vaccines are safe	619	3.70 (0.52)	0.93	-0.11
Vaccines are good	620	3.81 (0.45)	0.88	-0.27
Vaccines protect against diseases	619	3.66 (0.56)	0.77	0.17
Confident in ability to take child for vaccination	619	3.67 (0.59)	0.73	0.18
Community values vaccination services	618	3.50 (0.75)	0.71	0.18
Spouse or partner approves of vaccination	614	3.61 (0.70)	0.70	0.17
Measles is a health threat if unvaccinated	619	3.70 (0.58)	0.64	0.17
Illnesses that vaccine prevent are severe	618	3.68 (0.58)	0.60	0.28
Other parents approve of vaccination	615	3.55 (0.71)	0.60	0.29
How people talk about vaccination	569	2.64 (0.51)	0.49	0.24
Religion influences vaccination decision	618	3.31 (0.96)	-0.04	0.93
Vaccination is compatible with religious beliefs	613	3.35 (0.96)	0.01	0.92

*Number of responses and their unweighted item means

†Principal components factor extraction with an oblique Promax rotation to account for correlated factors

Table S2. Suitability of factor analysis and internal consistency of the 12-item Childhood Vaccination Acceptance and Confidence Scale–Sierra Leone, 2019

	12-item scale, Sierra Leone		
	Full scale	Sub-scale	
		Confidence	Congruence with faith
**Suitability of factor analysis**			
Kaiser-Myer-Olkin measure	0.93	-	-
Bartlett sphericity test; χ^2 *P*-value	<0.001	-	-
**Internal consistency**			
Cronbach's α (95%CI)	0.94	0.94 (0.93-0.95)	0.88 (0.85-0.91)

CI – confidence interval

Table S3. Association between vaccination confidence score and having a fully vaccinated child–Sierra Leone, 2019

	Fully vaccinated child*	
	**Adjusted PR† (95%CI)**	***P*-value‡**
**Confidence score**	1.26 (1.06-1.49)	0.01
**Child's place of delivery**		
Home	Reference	
Health facility	1.17 (0.96-1.43)	0.12
**Child's maternal birth order**		
First	Reference	
Second	1.00 (0.86-1.17)	0.97
Third or greater	1.02 (0.88-1.19)	0.75
**Parents' education**		
No parent has any education	Reference	
One parent has some education	1.16 (0.99-1.35)	0.06
Both parents have some education	1.23 (1.06-1.43)	0.01
**Parents' religion**		
Both Christian	Reference	
Both Muslim	0.97 (0.83-1.12)	0.66
Mixed faith	1.19 (1.03-1.36)	0.02
**Retention of child's vaccination card**		
Not retained	Reference	
Retained	1.74 (1.41-2.15)	<0.01

PR – adjusted prevalence ratio; CI – confidence interval

*For analytical purposes, 'fully vaccinated' was defined as receiving Bacillus Calmette-Guerin vaccine against tuberculosis; the first, second, and third dose of pentavalent vaccine against diphtheria, tetanus, pertussis, hepatitis B and Haemophilus influenzae type b; and first dose of measles-containing vaccine.

†Adjusted prevalence ratio from modified Poisson regression model with robust variance estimation by using generalized estimating equation; models adjusted for child's place of delivery, child's birth order, parents' education, parents' religion, retention of vaccination card.

‡Due to the complex sampling design, to get a valid p-value, the uncorrected χ^2 statistic was converted to an F statistic.

000	General Survey Information		
ID	QUESTION	RESPONSE	NOTES TO INTERVIEWER
1.	Region		*Select from list in ODK*
2.	District		*Select from list in ODK*
3.	Enumeration Area / Cluster ID	|____| |____|	*Select from list in ODK*
4.	Household ID	|____| |____| |____|	
5.	Household GPS coordinates		*Automatic detection in ODK*
6.	Date of interview	____ ____ / ____ ____ / 2019<br>MM DD	
7.	Team		*Select from list in ODK*
8.	Name of interviewer		*Select from list in ODK*
9.	Interviewer initials		*Enter in tablet*
10.	Attempted visit to this household	☐ First<br>☐ Second	
11.	Is the household head or another household member 15 years of age or older available at the time of the visit?	☐ 1. Yes<br>☐ 2. No-------------------------------→	END (age ineligibility): Thank the respondent for their time and schedule a revisit when primary caregiver over age 15 years will be available and continue to the next household.

100	Consent and Eligibility

Informed Consent Statement

Thank you for your time. My name is:_______. We are here today representing Sierra Leone Ministry of Health and its partners. We would like to ask you about your experience with immunizations that your child may have received. The information you provide will help the Ministry improve the country's childhood immunization programme. There are no right or wrong answers. We therefore ask you to speak candidly about your views and experiences. If you are not comfortable answering any of the questions, you do not have to do so. The information we collect in this interview will be anonymous, which means that you and your child/children will not be personally identified in any written reports. Participation in the survey is voluntary, and you can stop at any time for any reason. Because your views are important, we hope you will answer as many questions as possible. The interview should take about 45 minutes to conduct. Do you have any questions or comments for me at this point?

ID	QUESTION	RESPONSE	NOTES / SKIP PATTERN
101.	Do you consent to participate in the interview?	☐1. Yes<br>☐2. No-----------------------------------→	If NO, thank the person for his/her time and continue to the next household.
102.	How many children 12-23 months of age live in this household?	|____| |____| <br>*Enter 88 if don't know*<br>*Enter 99 if declined to answer*	If the interviewee doesn't know the number of children in the household enter 88 or if they decline to answer enter 99. Then ask to schedule a time to revisit when someone who knows and able to answer is available.
103.	Write the name of each child age 12-23 months of age in the box to the right.	1. Name:<br>2. Name:<br>3. Name:<br>4. Name:<br>5. Name:	If 2 or more children aged 12-23 months old live in the household, randomly select one of them. Write down the names on question 103. Using the Random Number Generator App installed on the tablet, randomly select a number between 1 and the total number of children aged 12-23 months in the household. The number selected will then correspond to the name listed next to that number, so that child will be the focus of this interview.

104.	What is your relationship with the selected child?	☐1. Mother<br>☐2. Father<br>☐3. Grandmother<br>☐4. Grandfather<br>☐5. Sibling<br>☐6. Other relative (specify:)<br>☐7. Other non-relative (specify)	
105.	Are you the primary caregiver of this child?	☐1. Yes-----------------------------------→<br>☐2. No	Q107
106.	Are you able to answer questions about this child's health on behalf of the caregiver?	☐1. Yes<br>☐2. No------------------------------------→	END (not able to answer questions about child) - If NO, thank the respondent for their time and schedule a revisit when primary caregiver will be available and continue to the next household.
107.	Do you know this child's date of birth?	☐1. Yes<br>☐2. No-----------------------------------→	If month or year is unknown, child is INELIGIBLE.<br><br>END: If there are no other eligible children in household, thank the respondent and continue to the next household.
108.	Date of birth of selected child	Day (DD) |____| |____| <br>Month (MM) |____| |____| <br>☐ 2017<br>☐ 2018	

200	**Demographic & Background Characteristics**<br>I would now like to ask you a few questions about your home.<br>Note: Ask child's name and substitute "this child" or "the child" with the name of the child in this section.		
ID	QUESTION	RESPONSE	GO TO
201.	How many people live in this household as primary residents?<br><br>*(defined as those who regularly sleep in under the same roof, eat from the same pot, and live together for the majority of the past year)*	|____| |____| <br>*Enter 88 if don't know*<br>*Enter 99 if declined to answer*	
202.	How many living children does this child's mother have in total?	|____| |____| children<br><br>*Enter 88 if don't know*<br>*Enter 99 if declined to answer*	
203.	Starting from the oldest child, what birth order is this child?	|____| |____| rank<br><br>*Enter 88 if don't know*<br>*Enter 99 if declined to answer*	
204.	What is this child's sex?	☐1. Female<br>☐2. Male	
205.	How long has this child's primary caregiver lived in this community?	|____| |____| years<br><br>or if less than 1 year:<br>|____| |____| months<br><br>*Enter 88 if don't know*<br>*Enter 99 if declined to answer*	
206.	What is the marital status of this child's mother?	☐1. Married or living together<br>☐2. Divorced or separated<br>☐3. Widowed<br>☐4. Never married and never lived together<br>☐88. Don't know<br>☐99. Declined to answer	
207.	What is the age of this child's mother?	|____| |____| Years<br><br>*Enter 88 if don't know*<br>*Enter 99 if declined to answer*	

208.	What is the highest level of school attended by this child's mother?	☐1. Never attended school<br>☐2. Primary<br>☐3. Junior secondary<br>☐4. Senior secondary<br>☐5. Vocational / tertiary<br>☐6. Higher<br>☐88. Don't know<br>☐99. Declined to answer	
209.	What is the highest level of school attended by this child's father?	☐1. Never attended school<br>☐2. Primary<br>☐3. Junior secondary<br>☐4. Senior secondary<br>☐5. Vocational / tertiary<br>☐6. Higher<br>☐88. Don't know<br>☐99. Declined to answer	
210.	What is the religion of this child's mother?	☐1. Christian<br>☐2. Muslim<br>☐3. Traditionalist<br>☐4. None<br>☐5. Other (specify)<br>☐88. Don't know<br>☐99. Declined to answer	
211.	What is the religion of this child's father?	☐1. Christian<br>☐2. Muslim<br>☐3. Traditionalist<br>☐4. None<br>☐5. Other (specify)<br>☐88. Don't know<br>☐99. Declined to answer	
212.	What does this child's mother do to earn money?	☐1. Private business (excluding petty traders)<br>☐2. Plumber/Carpenter/Electrician<br>☐3. Petty trader<br>☐4. Farmer<br>☐5. Teacher/lecturer/Instructor<br>☐6. Public transportation driver (taxi, buses, podapoda)<br>☐7. Okada driver<br>☐ 8. Medical or heath professional<br>☐ 9. Other government employee (not already listed)<br>☐ 10. Student<br>☐ 11. Unemployed<br>☐ 12. Retired<br>☐ 13. Other (specify)	

213.	What does this child's father do to earn money?	☐1. Private business (excluding petty traders)<br>☐2. Plumber/Carpenter/Electrician<br>☐3. Petty trader<br>☐4. Farmer<br>☐5. Teacher/lecturer/Instructor<br>☐6. Public transportation driver (taxi, buses, podapoda)<br>☐7. Okada driver<br>☐ 8. Medical or heath professional<br>☐ 9. Other government employee (not already listed)<br>☐ 10. Student<br>☐ 11. Unemployed<br>☐ 12. Retired<br>☐ 12. Other (specify)	
214.	How many times did the mother go for antenatal care (ANC) services when she was pregnant with this child?	|____| |____| *Enter 0 if never attended*<br>*Enter 88 if don't know*<br>*Enter 99 if declined to answer*	
215.	Where was the child born?	☐1. Home<br>☐2. Health facility (any type)<br>☐3. Traditional birth attendant (TBA) site<br>☐88. Don't know<br>☐99. Declined to answer	

The next several questions (301-313) are about your opinion on vaccination. For every question that I ask, please look at this figure. The biggest circle means “very much”, the second circle means “somewhat”, the small circle means “very little” and no circle means “not at all”. Please point to the circle that relates to how much you feel about every question.

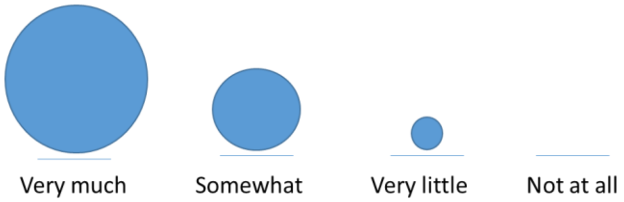

300	Vaccination Acceptance and Demand Part 1 – Attitudes & Beliefs		
ID	QUESTION	RESPONSE	GO TO
301.	How much do you think that vaccines are good for your child?	☐ 1. Not at all<br>☐ 2. Very little<br>☐ 3. Somewhat<br>☐ 4. Very much<br><br>☐ 99. Declined to answer	
302.	How much do you think that vaccines are safe for your child?	☐ 1. Not at all<br>☐ 2. Very little<br>☐ 3. Somewhat<br>☐ 4. Very much<br><br>☐ 99. Declined to answer	
303.	How much do you think that vaccines protect your child against diseases?	☐ 1. Not at all<br>☐ 2. Very little<br>☐ 3. Somewhat<br>☐ 4. Very much<br><br>☐ 99. Declined to answer	
304.	How much do you feel confident in your ability to take your child for scheduled vaccination visits?	☐ 1. Not at all<br>☐ 2. Very little<br>☐ 3. Somewhat<br>☐ 4. Very much<br><br>☐ 99. Declined to answer	
305.	How much would you encourage others to get their children vaccinated?	☐ 1. Not at all<br>☐ 2. Very little<br>☐ 3. Somewhat<br>☐ 4. Very much<br><br>☐ 99. Declined to answer	

306.	How much do people in your community value childhood vaccination services?	☐ 1. Not at all<br>☐ 2. Very little<br>☐ 3. Somewhat<br>☐ 4. Very much<br><br>☐ 99. Declined to answer	
307.	How much does your spouse or partner approve of childhood vaccination?	☐ 1. Not at all<br>☐ 2. Very little<br>☐ 3. Somewhat<br>☐ 4. Very much<br><br>☐ 99. Declined to answer	
308.	How much do other parents in your community approve of childhood vaccination?	☐ 1. Not at all<br>☐ 2. Very little<br>☐ 3. Somewhat<br>☐ 4. Very much<br><br>☐ 99. Declined to answer	
309.	How much do trusted leaders in your community approve of childhood vaccination?	☐ 1. Not at all<br>☐ 2. Very little<br>☐ 3. Somewhat<br>☐ 4. Very much<br><br>☐ 99. Declined to answer	
310.	How much does your religion influence vaccination decision for your child?	☐ 1. Not at all<br>☐ 2. Very little<br>☐ 3. Somewhat<br>☐ 4. Very much<br><br>☐ 99. Declined to answer	
311.	How much would you say childhood vaccination goes together with your religious beliefs?	☐ 1. Not at all<br>☐ 2. Very little<br>☐ 3. Somewhat<br>☐ 4. Very much<br><br>☐ 99. Declined to answer	
312.	How much of a health threat do you think measles is for children who are unvaccinated?	☐ 1. Not at all<br>☐ 2. Very little<br>☐ 3. Somewhat<br>☐ 4. Very much<br><br>☐ 99. Declined to answer	

313.	How much do you think the illnesses which vaccination prevent are severe?	☐ 1. Not at all<br>☐ 2. Very little<br>☐ 3. Somewhat<br>☐ 4. Very much<br><br>☐ 99. Declined to answer	

<<***Note to Interviewer***: *For questions in this section, you should read the question and say what the response options are – **except for questions Q409 and Q411*** >>

400	Vaccination Acceptance and Demand Part 2 - Experiences and Behaviors		
ID	QUESTION	RESPONSE	GO TO
401	Do you know of any child in your family or community that was infected with measles in the last year?	☐ 1. Yes<br>☐ 2. Unsure<br>☐ 3. No<br><br>☐ 99. Declined to answer	
402	Do you know of any child in your family or community that has experienced vaccine side effects in the last year?	☐ 1. Yes------→<br>☐ 2. Unsure------→<br>☐ 3. No ------→<br><br>☐ 99. Declined to answer	Q403<br>Q404<br>Q404
403	How serious was the vaccine side effect?	☐ 1. Not at all serious<br>☐ 2. A little serious<br>☐ 3. Somewhat serious<br>☐ 4. Very serious<br><br>☐ 99. Declined to answer	
404	How many vaccines do you find acceptable for your child to receive at a single visit?	☐ 1. One<br>☐ 2. Two<br>☐ 3. Three or more<br><br>☐ 99. Declined to answer	
405	During your last vaccination visit for this child, were you treated with respect by the vaccination staff?	☐ 1. Yes<br>☐ 2. Unsure<br>☐ 3. No<br><br>☐ 99. Declined to answer	

406	During you last vaccination visit for this child, were you satisfied with how the child was handled by the vaccination staff?	☐ 1. Yes<br>☐ 2. Unsure<br>☐ 3. No<br><br>☐ 99. Declined to answer	
407	How do people in your community usually speak about childhood vaccination services?	☐ 1. Positively<br>☐ 2. Mixed<br>☐ 3. Negatively<br><br>☐ 88. People don't speak about vaccination<br>☐ 99. Declined to answer	
408	Have you ever refused getting a recommended vaccine for your child?	☐ 1. Yes-------→<br>☐ 2. Unsure-------→<br>☐ 3. No -------→<br><br>☐ 99. Declined to answer -------→	Q409<br>Q410<br>Q410<br><br>Q410
409	What was the reason for refusing the recommended vaccine(s) for your child?<br><br>***(Do not read response options; multiple selections allowed)***	☐ 1. Lack of trust in vaccine<br>☐ 2. Lack of trust in vaccinator<br>☐ 3. Lack of trust in health system<br>☐ 4. Administering too many vaccines in the same visit<br>☐ 5. Fear of vaccination side effects<br>☐ 6. Long waiting time at vaccination site<br>☐ 7. Long distance to get to vaccination site<br>☐ 8. Lack of time to take the child<br>☐ 9. Concerns about cost<br>☐ 10. Religious concerns<br>☐ 11. Fertility concerns<br>☐ 12. Lack of benefit to vaccinate<br>☐ 13. Child was ill or had allergy<br>☐ 14. Other (specify)<br>☐ 15. No particular reasons<br><br>☐ 88. Don't remember<br>☐ 99. Declined to answer	
410	Have you ever delayed getting a recommended vaccine for your child?	☐ 1. Yes-------→<br>☐ 2. Unsure-------→<br>☐ 3. No -------→<br><br>☐ 99. Declined to answer -------→	Q411<br>Q412<br>Q412<br><br>Q412

411	What was the reason for delaying the recommended vaccine(s) for your child?<br><br>***(Do not read response options; multiple selections allowed)***	☐ 1. Lack of trust in vaccine<br>☐ 2. Lack of trust in vaccinator<br>☐ 3. Lack of trust in health system<br>☐ 4. Administering too many vaccines in the same visit<br>☐ 5. Fear of vaccination side effects<br>☐ 6. Long waiting time at vaccination site<br>☐ 7. Long distance to get to vaccination site<br>☐ 8. Lack of time to take the child<br>☐ 9. Concerns about cost<br>☐ 10. Religious concerns<br>☐ 11. Fertility concerns<br>☐ 12. Lack of benefit to vaccinate<br>☐ 13. Child was ill or had allergy<br>☐ 14. Other (specify)<br>☐ 15. No particular reasons<br><br>☐ 88. Don't remember<br>☐ 99. Declined to answer	
412	If you have another child in the future, do you plan to accept ALL recommended vaccinations for him/her?	☐ 1. Yes<br>☐ 2. Unsure<br>☐ 3. No<br><br>☐ 99. Declined to answer	

For the next set of questions (501-506) we would like to learn more about your social support, trusted information sources, and preferred ways of getting information about childhood vaccination services

500	Social support, trusted information sources and preferred communication channels		
ID	QUESTION	RESPONSE	GO TO
501	The last time the child was vaccinated, who took him/her to the vaccination site?<br><br>*(multiple selections allowed; do not read)*	☐1. Mother<br>☐2. Father<br>☐3. Grandmother<br>☐4. Grandfather<br>☐5. Sibling<br>☐6. Other relative (specify:)<br>☐7. Other non-relative (specify)<br><br>☐ 66. Vaccinated at home during outreach<br>☐ 77. Child has never been vaccinated<br>☐ 88. Don't remember / unsure<br>☐ 99. Declined to answer	
502	In your family, who has the final say over whether or not the child should receive vaccination?	☐1. Mother<br>☐2. Father<br>☐3. Both parents<br>☐4. Grandmother<br>☐5. Grandfather	

	(Do not read response options, select only one)	☐7. Other relative or family member (specify)<br>☐8. Other non-relative (specify)<br><br>☐ 88. Don't know<br>☐ 99. Declined to answer	
503	Which of the following means do you prefer the most to receive information about childhood vaccination?<br><br>*(Read all response options; ask respondent to select one)*	☐1. Household visits<br>☐2. Community-based events<br>☐3. Radio programming<br>☐4. Television programming<br>☐5. Social media (e.g. Facebook)<br>☐6. Mosque / church<br>☐7. Health facility (any type)<br>☐8. Pharmacy<br>☐9. Text messages<br>☐10. Voice messaging<br>☐11. Other (specify)<br>☐ 77. I don't have a preference<br>☐ 88. Don't know<br>☐ 99. Declined to answer	
504	Who do you trust the most to talk to you about childhood vaccination?<br>*(Do not read response options, select only one)*	☐1. Spouse<br>☐2. Child's grandparents<br>☐3. Other relatives<br>☐4. Friends and neighbors<br>☐5. Other parents and caregivers<br>☐6. Community leader (e.g. chief, village leader)<br>☐7. Faith leader<br>☐8. Traditional birth attendant (TBA)<br>☐9. Traditional healer<br>☐10. Community health worker<br>☐11. Health provider (e.g. nurse, doctor, vaccinator)<br>☐12. Pharmacist<br>☐13. Other (specify)<br>☐ 77. Don't trust anyone<br><br>☐ 88. Don't know<br>☐ 99. Declined to answer	
505	From your experience, what would you say is the most important area of improvement for childhood vaccination services in your community?<br><br>*(Do not read response options, select only one)*	☐1. More vaccination sites closer to the community<br>☐2. Offer vaccination services on weekends<br>☐3. Reduce the waiting time at vaccination sites<br>☐4. Make vaccines safer<br>☐5. Improve vaccinator interactions with caregivers<br>☐6. Enhance community engagement and education<br>☐7. Other (specify)<br>☐ 8. No improvements needed<br><br>☐ 88. Don't know<br>☐ 99. Declined to answer	

506	From your observation, how would you rate the level of community involvement and participation in the planning of vaccination programs in your community?	☐1. Low<br>☐2. Medium<br>☐3. High<br><br>☐ 88. Don't know<br>☐ 99. Declined to answer	

For the next set of questions (601-626) we would like to learn more about the ways in you have been reached with information and community engagements on childhood vaccination. We encourage you again to answer honestly to all questions as there are no right or wrong responses. If you have not been reached by certain interventions then you should feel free to say so. Only say yes to instances when you were directly reached and engaged.

600	Evaluation of vaccination demand promotion activities		
ID	QUESTION	RESPONSE	GO TO
601	In the past year, were you ever visited by a *community health worker*?	☐1. Yes--→<br>☐2. Unsure--→<br>☐3. No --→<br><br>☐ 99. Declined to answer---------------------------------→	Q602<br>Q605<br>Q605<br><br>Q605
602	Did the community health worker ever discuss vaccination during the visit?	☐1. Yes--→<br>☐2. Unsure--→<br>☐3. No --→<br><br>☐ 99. Declined to answer---------------------------------→	Q603<br>Q605<br>Q605<br><br>Q605
603	Did you trust the vaccination message by the community health worker?	☐1. Yes<br>☐2. Unsure<br>☐3. No<br><br>☐ 99. Declined to answer	
604	Did the vaccination message provided by the health worker during the household visit encourage you to get your child vaccinated?	☐1. Yes<br>☐2. Unsure<br>☐3. No<br><br>☐ 99. Declined to answer	
605	In the past year, was there any *organized community events* to discuss childhood vaccination in your community?	☐1. Yes--→<br>☐2. Unsure--→<br>☐3. No --→<br><br>☐ 99. Declined to answer---------------------------------→	Q606<br>Q609<br>Q609<br><br>Q609

606	Did you attend one or more of these organized community event(s)?	☐1. Yes---→<br>☐2. Unsure--→<br>☐3. No --→<br><br>☐ 99. Declined to answer---------------------------------→	Q607<br>Q609<br>Q609<br><br>Q609
607	Did you trust the vaccination message at these organized community event(s)?	☐1. Yes<br>☐2. Unsure<br>☐3. No<br><br>☐ 99. Declined to answer	
608	Did the vaccination message during the organized community event(s) encourage you to get your child vaccinated?	☐1. Yes<br>☐2. Unsure<br>☐3. No<br><br>☐ 99. Declined to answer	
609	In the past year, did your ***faith leader*** promote childhood vaccination through a sermon or personal counsel?	☐1. Yes---→<br>☐2. Unsure--→<br>☐3. No --→<br><br>☐ 99. Declined to answer---------------------------------→	Q610<br>Q612<br>Q612<br><br>Q612
610	Did you trust the vaccination message from your faith leader?	☐1. Yes<br>☐2. Unsure<br>☐3. No<br><br>☐ 99. Declined to answer	
611	Did the vaccination message from your faith leader encourage you to get demand your child vaccinated?	☐1. Yes<br>☐2. Unsure<br>☐3. No<br><br>☐ 99. Declined to answer	
612	In the past year, did you listen to any ***radio programs*** that discussed childhood vaccination?	☐1. Yes---→<br>☐2. Unsure--→<br>☐3. No --→<br><br>☐ 99. Declined to answer---------------------------------→	Q613<br>Q615<br>Q615<br><br>Q615
613	Did you trust the vaccination message during the radio program(s)?	☐1. Yes<br>☐2. Unsure	

		☐3. No<br><br>☐ 99. Declined to answer	
614	Did the vaccination message during the radio program(s) encourage you to get your child vaccinated	☐1. Yes<br>☐2. Unsure<br>☐2. No<br><br>☐ 99. Declined to answer	
615	In the past year, did you watch any ***television programs*** that discussed childhood vaccination?	☐1. Yes--→<br>☐2. Unsure--→<br>☐3. No --→<br><br>☐ 99. Declined to answer---------------------------------→	Q616<br>Q618<br>Q618<br><br>Q618
616	Did you trust the vaccination message during the television program(s)?	☐1. Yes<br>☐2. Unsure<br>☐3. No<br><br>☐ 99. Declined to answer	
617	Did the vaccination message during the television program(s) encourage you to get your child vaccinated?	☐1. Yes<br>☐2. Unsure<br>☐3. No<br><br>☐ 99. Declined to answer	
618	In the past year, did you see any **social media** messages that discussed childhood vaccination?	☐1. Yes--→<br>☐2. Unsure--→<br>☐3. No --→<br><br>☐ 99. Declined to answer---------------------------------→	Q619<br>Q621<br>Q621<br><br>Q621
619	Did you trust the vaccination message on social media platform(s)?	☐1. Yes<br>☐2. Unsure<br>☐3. No<br><br>☐ 99. Declined to answer	
620	Did the vaccination message during the social media platform(s) encourage you to get your child vaccinated?	☐1. Yes<br>☐2. Unsure<br>☐3. No<br><br>☐ 99. Declined to answer	

621	In the past year, did you see any **printed materials** (such as posters and banners) that contained information about childhood vaccination?	☐1. Yes--→<br>☐2. Unsure---→<br>☐3. No --→<br><br>☐ 99. Declined to answer---------------------------------→	Q622<br>Q624<br>Q624<br><br>Q624
622	Did you trust the vaccination message in the printed material(s)?	☐1. Yes<br>☐2. Unsure<br>☐3. No<br><br>☐ 99. Declined to answer	
623	Did the vaccination message in the printed material(s) encourage you to get your child vaccinated?	☐1. Yes<br>☐2. Unsure<br>☐3. No<br><br>☐ 99. Declined to answer	
624	In the past year, did you visit any **health facility** where you received information about childhood vaccination services?	☐1. Yes--→<br>☐2. Unsure---→<br>☐3. No --→<br><br>☐ 99. Declined to answer---------------------------------→	Q625<br>Q701<br>Q701<br><br>Q701
625	Did you trust the vaccination message during the health facility visit(s)?	☐1. Yes<br>☐2. Unsure<br>☐3. No<br><br>☐ 99. Declined to answer	
626	Did the vaccination message during the health facility visit(s) encourage you to get your child vaccinated?	☐1. Yes<br>☐2. Unsure<br>☐3. No<br><br>☐ 99. Declined to answer	

I will now ask you few questions about Measles and Rubella.

700	Measles-Rubella Vaccine Awareness and Acceptance		
ID	QUESTION	RESPONSE	GO TO
701	Have you heard about Measles?	☐1. Yes<br><br>☐2. No	

702	Have you heard about Rubella?	☐1. Yes<br>☐2. No	

Vignette:
RUBELLA is a contagious disease caused by a virus. Most people who get rubella usually have mild illness, with symptoms that can include a low-grade fever, sore throat, and a rash that starts on the face and spreads to the rest of the body. Some people may also have a headache, pink eye, and general discomfort before the rash appears. Rubella can cause a miscarriage or serious birth defects in an unborn baby if a woman is infected while she is pregnant.

MEASLES is a very contagious disease caused by a virus. It spreads through the air when an infected person coughs or sneezes. Measles starts with fever. Soon after, it causes a cough, runny nose, and red eyes. Then a rash of tiny, red spots breaks out. It starts at the head and spreads to the rest of the body.

703	If offered to your child, would you accept one vaccine that prevents both Measles and Rubella?	☐1. Yes--→<br>☐2. Unsure --→<br>☐3. No--→<br>☐ 99. Declined to answer ---------------------------------→	801<br>704<br>704<br>801
704	What is the primary reason you would not accept or are unsure of accepting the vaccine?<br><br>*(do not read options; select one)*	☐1. MR vaccine is not safe<br>☐2. MR vaccine reduces fertility<br>☐3. MR vaccine is not halal<br>☐4. No particular reason<br>☐5. Other (specify):<br><br>☐88. Don’t know<br>☐99. Declined to answer	

We are almost at the end of the interview. Let's talk briefly about the child's vaccination history.

800	Child's Vaccination History		
ID	QUESTION	RESPONSE	GO TO
801	Do you have an immunization card [yellow card] or a card where the child's vaccinations are written down?<br><br>**If YES, May I see it please?**	☐1. Yes, seen---→<br>☐2. Yes, not seen ---→<br>☐3. No card --→	Q803<br>Q802<br>Q802
802	What is the reason for not having an immunization card [yellow card] or other documentation of immunization history for this child?<br><br>***Do not read responses***<br><br>*Ask for the main reason and mark ONLY ONE.*	☐1. Child never received an immunization card<br>☐2. Health facility keeps the immunization card<br>☐3. Immunization card is lost or destroyed<br>☐4. Immunization card is kept at a different location<br>☐5. Other (Specify:________________________)<br><br>☐88. Don't know<br>☐99. Declined to answer	
803	As far as you know, has your child received all of the recommended immunizations up to the current age of the child?	☐1. Yes<br>☐2. No<br><br>☐88. Don't know<br>☐99. Declined to answer	
804	How old was the child (in months) at his/her most recent vaccination visit?	☐☐ months<br><br>Enter 99 if never vaccinated ---------------------------------→	Q1000

Request to see the health card / immunization card of the infant.<br>For each vaccine, check « Yes » if there is a checkmark or a date for the vaccine and « no » if there is nothing written.<br>Copy the dates. Write « 99 », if there is a poorly written or missing day/month and « 9999 » for missing year.<br><br>*IF CARD NOT AVAILABLE SKIP TO SECTION 900*					
805	Child's date of birth (DOB) recorded on card ☐☐ / ☐☐ / ☐☐☐☐<br>a. DD b. MM c. YYYY				
806	Type of card(s)<br><br>***Mark all available.***		☐1. Child Health Record Book<br>☐2. Yellow Card<br>☐3. Piece of paper/other documentation		
	Vaccine	a. Vaccine received	b. Day	c. Month	d. Year
807	BCG	☐1. Yes<br>☐2. No	☐☐	☐☐	☐☐☐☐
808	Pentavalent (1)<br>DTP/Hib/HepB	☐1. Yes<br>☐2. No	☐☐	☐☐	☐☐☐☐
809	Pentavalent (2)<br>DTP/Hib/HepB	☐1. Yes<br>☐2. No	☐☐	☐☐	☐☐☐☐
810	Pentavalent (3)<br>DTP/Hib/HepB	☐1. Yes<br>☐2. No	☐☐	☐☐	☐☐☐☐
811	Measles (1)	☐1. Yes<br>☐2. No	☐☐	☐☐	☐☐☐☐
812	Measles (2)	☐1. Yes<br>☐2. No	☐☐	☐☐	☐☐☐☐

813	Insecticide treated bednet received	☐1. Yes<br>☐2. No<br>☐3. Info not recorded	☐☐	☐☐	☐☐☐☐

900	Recall of Child's Vaccination History<br>*Only when vaccination card not available*		
ID	QUESTION	RESPONSE	GO TO
901	Has the child ever received an injection in the right upper arm or shoulder that usually causes a scar? – that is, BCG vaccination (against tuberculosis). **This is usually given at birth or very shortly after.**	☐1. Yes-------→<br>☐2. No-------→<br>☐88. Don't know-------→<br>☐99. Declined to answer -------→	Q902<br>Q903<br>Q903<br>Q903
902	If the child is present, check for evidence of a scar and record	☐1. Scar Present<br>☐2. No Scar Present<br>☐3. Child not available to check	
903	Has the child ever received an injection on the left thigh? – that is a five-in-one vaccination (pentavalent) to prevent him/her from getting tetanus, whooping cough, diphtheria, influenza & hepatitis. **It is usually given to the child at 6 weeks, 10 weeks, and 14 weeks of age.**	☐1. Yes -------→<br>☐2. No -------→<br>☐88. Don't know-------→<br>☐99. Declined to answer -------→	Q904<br>Q905<br>Q905<br>Q905
904	How many times did the child receive pentavalent vaccine on the left thigh?	Number of times: __________<br>*Enter 88 if don't remember / unsure*<br>*Enter 99 if declined to answer*	
905	Has the child ever received an injection on the **upper arm**? that is measles injection at the **age of 9 months or older** - to prevent him/her from getting measles	☐1. Yes -------→<br>☐2. No -------→<br>☐88. Don't know-------→<br>☐99. Declined to answer -------→	Q906<br>Q1001<br>Q1001<br>Q1001
906	How many times was measles vaccine given at a routine immunization session?	Number of times: ________	

This last section of the interview focuses on the accessibility of vaccination services in this community.

1000	Availability and Accessibility of Childhood Vaccination Services		
ID	QUESTION	RESPONSE	GO TO
1001	Approximately, how long does it take to get to the nearest site where childhood vaccines are provided?	_____ hour(s)<br>_____ minute(s)<br><br>*Enter 0 for hour if less than 1 hour*	
1002	How do you view the time it usually takes to reach your usual vaccination site?	☐1. Too much time<br>☐2. About right<br>☐3. A short time<br><br>☐99. Declined to answer	
1003	Approximately how long does it usually take to get the child vaccinated at the health facility or clinic?	_____ hour(s)<br>_____ minute(s)<br><br>*Enter 0 for hour if less than 1 hour*	
1004	How do you view the time it usually takes to get your child vaccinated once you arrive at the vaccination site?	☐1. Too much time<br>☐2. About right<br>☐3. A short time<br><br>☐99. Declined to answer	
1005	How much money are you usually expected to give to healthcare workers during a vaccination visit, if any?	☐1. Nothing---→<br>☐2. 1,000-5,000 Leones<br>☐3. 6,000-10,000 Leones<br>☐4. >10,000 Leones<br><br>☐88. Don't remember / unsure<br>☐99. Declined to answer	Q 1007
1006	Do you find the amount of money reasonable in order to get your child vaccinated?	☐1. Yes<br>☐2. Unsure<br>☐3. No<br><br>☐99. Declined to answer	
1007	Are you usually expected to provide any non-cash item to health workers in order to get your child vaccinated?	☐1. Yes<br>☐2. Unsure<br>☐3. No<br><br>☐99. Declined to answer	

1008	Do you find the non-cash item reasonable in order to get your child vaccinated?	☐1. Yes<br>☐2. Unsure<br>☐3. No<br><br>☐99. Declined to answer	
1009	Have you ever taken any child for vaccination and come home without receiving vaccination?	☐1. Yes--→<br>☐2. No--→<br><br>☐88. Don't remember --------------------------------------→<br>☐99. Declined to answer----------------------------------→	Q1010<br>Q1011<br><br>Q1011<br>Q1011
1010	What was the main reason your child did not receive vaccination the last time that happened (*referring to last question*)?	☐1. Vaccines were not available<br>☐2. Was not a scheduled vaccination day<br>☐3. Line was too long<br>☐4. I was asked for pay for service<br>☐5. I chose not to go through with vaccination<br>☐6. Other (specify:________________________)<br><br>☐88. Don't remember / unsure<br>☐99. Declined to answer	
1011	Where did this child receive their most recent immunization?	☐1. Hospital<br>☐2. Community Health Center (CHC)<br>☐3. Community Health Post (CHP)<br>☐4. Maternal and Child Health Post (MCHP)<br>☐5. At home during outreach<br><br>☐77. Child never vaccinated before<br>☐88. Don't know / don't remember<br>☐99. Declined to answer	
1012	If health facility, what type of facility?	☐1. Public / Government<br>☐2. Private (non-NGO)<br>☐3. NGO<br><br>☐88. Don't know / don't remember<br>☐99. Declined to answer	

**Outcome of visit**	Select one
Completed interview	1
Interview partially completed	2
Postponed	3
Refused to be interviewed	4
No eligible respondents in household	5
Cannot confirm child's age / eligibility	6
Dwelling not found	7
Other (specify):	8

END OF QUESTIONNAIRE:

Thank you for sharing your experiences and opinions relating to childhood vaccination in your community. The information you provided is valuable, and will help the Sierra Leone Ministry of Health and Sanitation improve childhood vaccination services.

Do you have any questions for me before we end the interview?

- *If YES----------------→Answer question(s)*
    - *if you don't know the answer or feel uncomfortable providing an answer, you should feel free to say so…then ask the respondent to visit the nearest health facility to get more information*

- *If NO---------------→END*
